# Assessing the Anti-Inflammatory Activity of the Anxiolytic Drug Buspirone Using CRISPR-Cas9 Gene Editing in LPS-Stimulated BV-2 Microglial Cells

**DOI:** 10.3390/cells10061312

**Published:** 2021-05-25

**Authors:** Sarah Thomas Broome, Teagan Fisher, Alen Faiz, Kevin A. Keay, Giuseppe Musumeci, Ghaith Al-Badri, Alessandro Castorina

**Affiliations:** 1 Laboratory of Cellular and Molecular Neuroscience (LCMN), School of Life Sciences, Faculty of Science, University of Technology Sydney, Sydney, NSW 2007, Australia; sarah.j.thomasbroome@student.uts.edu.au (S.T.B.); teagan.fisher@unsw.edu.au (T.F.); g.al-badri@unsw.edu.au (G.A.-B.); 2 Respiratory Bioinformatics and Molecular Biology (RBMB) Group, School of Life Sciences, Faculty of Science, University of Technology Sydney, Sydney, NSW 2007, Australia; alen.faiz@uts.edu.au; 3 Laboratory of Neural Structure and Function (LNSF), School of Medical Sciences (Neuroscience), Faculty of Medicine and Health, University of Sydney, Sydney, NSW 2006, Australia; kevin.keay@sydney.edu.au; 4 Section of Human Anatomy and Histology, Department of Biomedical and Biotechnological Sciences, University of Catania, via S. Sofia, 87, 95123 Catania, Italy; g.musumeci@unict.it

**Keywords:** microglia, dopamine D3 receptor, 5-hydroxytryptamine 1a receptor, neuroinflammation, Parkinson’s disease

## Abstract

Buspirone is an anxiolytic drug with robust serotonin receptor 1A (Htr1a) agonist activities. However, evidence has demonstrated that this drug also targets the dopamine D3 receptor (Drd3), where it acts as a potent antagonist. In vivo, Drd3 blockade is neuroprotective and reduces inflammation in models of Parkinson’s disease. To test if buspirone also elicited anti-inflammatory activities in vitro, we generated stable Drd3^−/−^ and Htr1a^−/−^ BV2 microglial cell lines using CRISPR-Cas9 technology and then tested the effects of buspirone after lipopolysaccharide (LPS) challenge. We found that LPS exposure had no effect on cell viability, except in Htr1a^−/−^ cells, where viability was reduced (*p* < 0.001). Drug treatment reduced viability in Drd3^−/−^ cells, but not in WT or Htr1a^−/−^ cells. Buspirone counteracted LPS-induced NO release, NOS2, IL-1β and TNF-α gene expression in WT cells, whereas it exerted limited effects in Drd3^−/−^ or Htr1a^−/−^ microglia. In summary, our findings indicate that buspirone attenuates microglial polarization after LPS challenge. These results also highlight some major effects of Drd3 or Htr1a genetic ablation on microglial biology, raising important questions on the complex role of neurotransmitters in regulating microglia functions.

## 1. Introduction

Buspirone (Buspar^®^) is a FDA-approved selective 5-hydroxytryptamine receptor (5-HT1Ar) partial agonist used in the treatment of generalised anxiety disorder [1]. It is well known that 5-hydroxytryptamine, or serotonin (5-HT) regulates both mood and sleep, and drugs that stimulate 5-HT activity are commonly used therapeutically for anxiety and depression [2,3]. However, it has also been shown that Buspirone has a strong “off-target” function as a dopamine-3-receptor (Drd3) antagonist [4,5,6].

Drd3’s are found mainly in the *striatum*, particularly within the *nucleus accumbens* where they are involved in the regulation of motor function, impulse control, memory performance, addiction and drug tolerance [6,7,8,9,10]. However, a novel role for dopamine receptors (DRs) in neuroinflammation has been revealed [11]. McKenna and colleagues, showed that functional DRs are expressed on the surface of multiple immune cell types including microglia [12].

Dopamine (DA) can exert its effects through interactions with any of the five DRs expressed on the membrane of target cells [13]. This is seen clearly in the ability of the Drd3 to promote inflammation [14]. For example, Elgueta et al. [15] showed that Drd3-signalling promotes the development of PD by favoring neuroinflammation and the pathogenic CD4+ T cell response associated with Parkinson’s disease (PD) [15]. Furthermore, studies using experimental compounds have shown that blocking the Drd3, significantly reduced inflammation and consequently slowed the progression of PD [11,15]. 

PD is a progressive neurodegenerative disorder characterised by a loss of dopaminergic neurons in the *substantia nigra pars compacta* resulting in diminished DA availability in the *striatum* [16]. PD is also associated with chronic neuroinflammation [17]. As such, the abnormal DA signalling observed in PD could contribute not only to altered DA neurotransmission, but also to an altered immune response. This is particularly important as evidence from studies of LPS-mediated neurotoxicity [18] has shown that inflammatory responses from non-neuronal cells alone are sufficient to cause loss of DAergic neurons. Injection of LPS into the rodent brain results in increased levels of inflammatory mediators, including cyclooxygenase-2 (COX-2) and inducible nitric oxide synthetase (iNOS), prior to the loss of DA-ergic neurons [19]. This suggests that inflammation may precede neurodegeneration, which ultimately results in PD. Accordingly, blocking Drd3-mediated signalling presents a promising targeted therapeutic strategy for PD.

Neuroinflammation describes the local inflammatory response within the brain and is driven predominantly by microglia, the specialised scavengers of the brain [20,21]. Normally, microglia exist in a resting state and contribute to immune surveillance [22]. In response to injury or pathological *stimuli*, microglia become activated and release cytotoxic and pro-inflammatory molecules [23], which have been shown to promote the degeneration of DA neurons [24]. This has been shown in several studies in which the chronic activation of microglia exacerbates neurodegeneration, as the excessive and sustained secretion of neurotoxic molecules such as nitric oxide (NO) and pro-inflammatory cytokines become detrimental to neighboring neuronal cells [24]. DA receptors expressed by microglia appear to modulate inflammatory responses and neuronal survival [25]. For example, it has been shown that microglial activation is prevented in Drd3-deficient mice, in the MPTP-(1-methyl-4-phenyl-1,2,3,6-tetrahydropyridine)-model of PD [15]. Furthermore, the anti-PD drug rasagiline, a brain-selective MAO inhibitor, significantly reduced both reactive oxygen species (ROS) and NO secretion, as well as cytokine release in DA stimulated microglia [26]. Collectively, these results suggest that DA can hamper the ability of microglia to secrete cytokines and shift towards activated phenotypes. 

Since microglia are the key drivers of neuroinflammation, it is crucial to determine how Drd3-mediated activity regulates these cells. For example, in PD it is well accepted that neuroinflammation drives progressive neurodegeneration. In fact, neuronal damage and uncontrolled inflammation amplify each other to induce a feed-forward vicious cycle driving the chronic progression of PD and other neurodegenerative diseases [27]. These observations underpin the substantial experimental efforts in exploring the potential utility of traditional anti-inflammatory drugs able to slow, reverse and prevent PD [11]. However, due to the peripheral effects of the long-term use of anti-inflammatory drugs, novel targets are needed specifically to counteract CNS inflammation. Studies using experimental compounds that block the Drd3 pharmacologically could help in determining whether targeting these receptors may provide a viable disease-modifying strategy to reduce chronic inflammation in PD, and perhaps for other neuroinflammatory disorders [11]. 

Buspirone could be one such drug. Therefore, in this study, we sought to investigate whether buspirone prevents inflammation in microglial cells exposed to the inflammatory mimetic LPS. To the best of our knowledge, no other studies have tested these effects in microglia. In the established BV-2 microglial cell line, we implemented CRISPR-Cas9 technology to generate two independent cell lines harboring a targeted Drd3 or Htr1a gene deletion, Buspirone’s best known pharmacological targets. We used a combination of molecular and cellular biological techniques to investigate: (i) the viability of these cells following gene deletion, and (ii) the functional consequences of gene deletions in response to the inflammatory mimetic, lipopolysaccharide (LPS).

## 2. Materials and Methods

### 2.1. Generation of Knockout Cell Lines Using CRISPR-Cas9 Gene Editing 

The CRISPR-Cas9 plasmid Px458 was used to generate knockout cell lines, as previously described [28]. Guide RNA (gRNA) sequences (summarised in Table 1) were generated using the online design tool Benchling and synthesised by Sigma-Aldrich (Castle Hill, NSW, Australia). Plasmids were cloned in DH5-α *Escherichia coli* and, after a single colony selection from ampicillin positive (100 µg/mL) plates, extracted using the GeneJET Plasmid Miniprep Kit (ThermoFisher Scientific, North Ryde, NSW, Australia). Transfection was carried out in a 12-well plate with a seeding density of 1 × 10^5^ cells per well. Cells were transfected in 3 µL of Lipofectamine 3000 (Invitrogen) diluted in 62.5 µL of Opti-MEM (ThermoFisher Scientific). This was combined with a DNA mixture containing 2 µg of purified plasmid, 4 µL of P3000 reagent (Invitrogen, Carlsbad, CA, USA) and 125 µL of Opti-MEM (Thermofisher Scientific). Cells were incubated in the mixture for 4 h at 37 °C with 5% CO_2_. After incubation, half of the mixture was removed and replaced with Opti-MEM and incubated for 24 h at 37 °C with 5% CO_2_. Transfected cells were then single-cell sorted into a 96-well plate using the MoFlo© Astrios Cell Sorter, gating for green fluorescent protein (GFP) positive cells. Single-cell-derived colonies (isogenic populations) were expanded for biochemical and molecular analysis. To identify the status of genome editing, genomic DNA was extracted from the isogenic populations using ISOLATE II Genomic DNA Kit (Bioline, NSW, Australia), and end-point PCR amplification was conducted with MyTaq DNA Polymerase Kit (Bioline), using primer sets described in Table 2. The amplification took place in the T100 Thermal Cycler (Bio-Rad, Gladesville, NSW, Australia) under the following instrument settings: (1) 95 °C for 60 s, (2) 95 °C for 15 s, (3) 56 °C for 15 s, (4) 72 °C for 10 s, (5) repeat step 2–4 for 35 cycles. Following end-point PCR, we confirmed that the primers amplified the correct product by Agarose Gel Electrophoresis (AGE) (Supplementary Figure S3). Following confirmation by AGE, PCR products were purified using SureClean Plus (Bioline) and sent to Australian Genome Research Facility (AGRF) for Sanger sequencing using the primers described in Table 3. Sanger sequencing results were analysed using online tools Benchling and Tracking of Indels by Decomposition: TIDE. 

### 2.2. Cell Culture 

Mouse microglial BV-2 cells were used in this study. These cells share a high transcriptome homology and response to inflammatory challenges with primary microglia [29]. Cells were grown in full growth media containing Dulbecco’s modified eagle’s medium nutrient mixture F-12 HAM (1:1 *vol*/*vol* DMEM/F12) (Sigma-Aldrich, Castle Hill, NSW, Australia), 10% heat-inactivated foetal bovine serum (FBS, Sigma-Aldrich, Castle Hill, NSW, Australia) and 1% penicillin/streptomycin solution (Sigma-Aldrich, Castle Hill, NSW, Australia) and were kept in an incubator with humidified air containing 5% CO_2_ at a temperature of 37 °C. Cells were treated with 1 µg/mL LPS (L4391, *Escherichia coli* 0111: B4, Sigma-Aldrich) and/or 300 µM buspirone (Sigma-Aldrich) for 24 h at 37 °C in a humidified atmosphere with 5% CO_2_. LPS concentrations and buspirone concentrations used in this study were based on preliminary observations (data not shown). 

### 2.3. Cell Viability

To assess cell viability, we used the Cell Proliferation Kit I (MTT) (Sigma-Aldrich). Cells were seeded at 2 × 10^4^ cells per well in a 96-well plate and incubated at 37 °C with 5% CO_2_ until cells reached 80% confluence. Cells were treated for 24 h with control media, 1 µg/mL LPS or both LPS and 300 µM buspirone. After 24 h, 10 µL of MTT labelling reagent was added to each well. After 4 h, 100 µL of the solubilization solution (10% SDS in 0.01 M HCl) was added to each well and incubated at 37 °C overnight. Absorbance was measured at 565 nm in the TECAN infinite M1000-PRO ELISA reader (ThermoFisher Scientific). 

### 2.4. Nitric Oxide (Griess Reagent Assay) 

Cells were seeded at 2 × 10^4^ cells per well in a 96-well plate and incubated at 37 °C with 5% CO_2_ until cells reached 80% confluence. Cells were treated for 24 h with control media, 1 µg/mL LPS or both LPS and 300 µM buspirone. The supernatant was collected and placed into a new 96-well plate. A total of 100 µL of freshly prepared Griess reagent was then added to each well and incubated at room temperature for 15 min on a slow oscillation protected from light. Absorbance was measured at 540 nm using the TECAN infinite M1000-PRO ELISA reader. Optical density values from each group were recorded and reported as a percentage of control. 

### 2.5. RNA Extraction and cDNA Synthesis

Total RNA was extracted using 1 mL TRI reagent (Sigma-Aldrich, Castle Hill, NSW, Australia) and 0.2 mL chloroform and precipitated with 0.5 mL 2-propanol (Sigma-Aldrich). Pellets were washed twice with 75% ethanol and air-dried. RNA concentrations were calculated using NanoDrop™ 2000 (ThermoFisher Scientific). A total of 1 µg of total RNA were loaded in each cDNA synthesis reaction. cDNA synthesis was conducted using the T1000 thermal cycler (Bio-Rad, Gladesville, NSW, Australia) in a final volume of 20 µL. Each reaction contained 1 μg of RNA diluted in a volume of 11 µL, to which we added 9 µL of cDNA synthesis mix (Tetro cDNA synthesis kit) (Bioline, Australia). Samples were incubated at 45 °C for 40 min followed by 85 °C for 5 min. Finally, cDNA samples were stored at −20 °C until use.

### 2.6. Real-Time Quantitative Polymerase Chain Reaction 

Real-time qPCR analyses were carried out as previously reported [30,31], with minor modifications. For each gene of interest, qPCRs were performed in a final volume of 10 μL, which comprised 3 μL cDNA, 0.4 μL milliQ water, 5 μL of iTaq Universal SYBR green master mix (BioRad, Gladesville, NSW, Australia) and 0.8 μL of the corresponding forward and reverse primers (5 μM, Sigma-Aldrich, Castle Hill, NSW, Australia) to obtain a final primer concentration of 400 nM. The primers are described in Table 4. Reaction mixtures were loaded in Hard-Shell^®^ 96-Well PCR Plates, and four genes of interest were tested in each run using the CFX96 Touch™ Real-Time PCR Detection System (Bio-Rad, Gladesville, NSW, Australia). Instrument settings were as follows: (1) 95 °C for 2 min, (2) 60 °C for 10 s, (3) 72 °C for 10 s, (4) plate read, (5) repeat step 2 to 4 for 45 cycles. For the melting curve analyses, settings were (1) 65 °C for 35 s, (2) plate read, (3) repeat step 1–2 for 60 times). To examine changes in expression, we analysed the mean fold change values of each sample, calculated using the ΔΔCt method, as previously described by Schmittgen and Livak [32]. PCR product specificity was evaluated by melting curve analysis, with each gene showing a single peak (data not shown). 

### 2.7. Protein Extraction and Western Blot

Proteins were extracted using radioimmunoprecipitation assay (RIPA) buffer (Sigma-Aldrich, Castle Hill, NSW, Australia) containing 1 × Protease Inhibitor cocktail (cOmplete™, Mini, EDTA-free Protease Inhibitor Cocktail, Sigma-Aldrich, Castle Hill, NSW, Australia). Protein was quantified using the Bicinchoninic-Acid (BCA) Assay Protein Assay Kit (ThermoFisher Scientific) according to manufacturer’s protocol and measured using the TECAN infinite M1000-PRO ELISA reader at 562 nm. 

Samples were prepared by adding 3.75 μL of Laemmli Buffer (Bio-Rad, Gladesville, NSW, Australia) containing β-mercaptoethanol (Sigma-Aldrich, Castle Hill, NSW, Australia) mixture, (ratio 1:9 *vol*/*vol*) to 30 μg protein in a final volume of 15 μL. Samples were then heated for 10 min at 70 °C to denature proteins. Proteins were then separated by SDS-polyacrylamide gel electrophoresis (SDS-PAGE) using 4–20% mini gels (Bio-Rad, Criterion 15-well Mini-Protean SFX), alongside 5 µL of the molecular weight ladder/marker (Bio-Rad Pre-stained HyperLadder Precision Plus Protein™). Gels were transferred to a PVDF membrane using the Trans-Blot Turbo instrument (Bio-Rad) [33]. Once terminated, membranes were immediately placed in a container filled with TBS +0.1% Tween 20 (Sigma-Aldrich, Castle Hill, NSW, Australia) (TBST 1×) to wash out any residues during transfer. To block non-specific binding sites, membranes were blocked for 1 h in 5% dry non-fat skim milk in TBST with slow agitation (50–60 rpm). 

Membranes were incubated with appropriately diluted primary antibodies in blocking buffer overnight at 4 °C with slow agitation. The following primary antibodies were used: GAPDH (VPA00187, Rb, 1:2000, BioRad, Gladesville, NSW, Australia); 5HT1ar (ab85615, Rb, 1:400, Abcam, Cambridge MA, USA). This was followed by incubation with host-specific secondary antibodies. Membranes were then placed in a container with 1 × TBST and washed rapidly three times, followed by three further 5 min washes. Finally, membranes were incubated in secondary antibody (HRP-conjugated goat anti-rabbit IgG) for 1 h at room temperature, diluted in blocking buffer. The membranes were then washed once again as previously described to remove excess secondary antibody [34]. Imaging was then performed on the Bio-Rad ChemiDoc MP Imaging System (Bio-Rad). To detect bands, we utilized Clarity Western ECL Blotting Substrate (Bio-Rad). Densitometric analyses of bands was computed using NIH ImageJ (https://imagej.nih.gov/ij/download/ (accessed on 14 January 2021)). Optical densities of target proteins were normalised to those of loading controls (GAPDH).

### 2.8. Statistical Analysis 

All data are reported as mean ± S.E.M. Statistical analyses were calculated using GraphPad Prism software ver. 8.4.1 (GraphPad Software, San Diego, California, CA, USA, www.graphpad.com, accessed on 14 January 2021). Comparisons between two groups were evaluated using the unpaired two-tailed Student’s *t*-test. Differences between three or more groups were analysed using one-way ANOVA and Tukey post-hoc test. To compute statistical comparisons that involved multiple independent variables, two-way ANOVA was used followed by Tukey post-hoc test. *p* values ≤ 0.05 were considered statistically significant. 

## 3. Results

### 3.1. Generation of Stable Drd3^−/−^ and Htr1a^−/−^ Cell Lines

**Figure 1:**
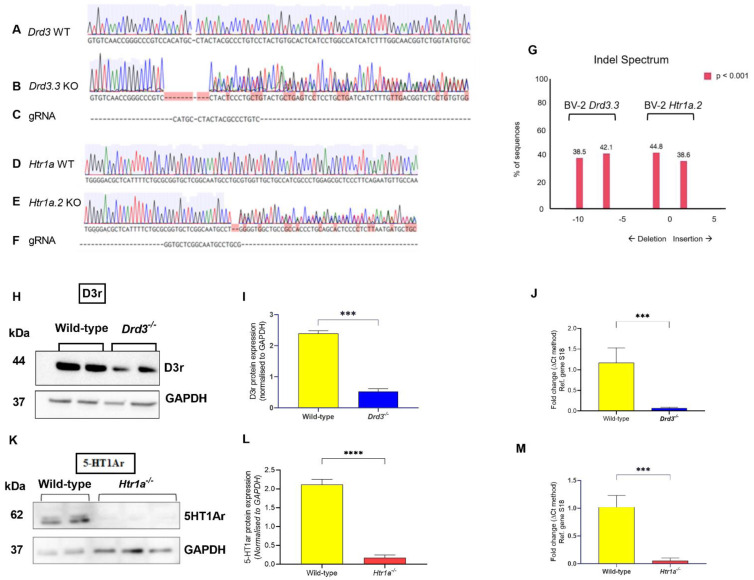
Analyses to confirm stable deletions of the *Drd3* or *Htr1a* genes in BV-2 microglial cells. PCR products of the *Drd3* and *Htr1a* genes in the WT, *Drd3*^−/−^ clones and *Htr1a*^−/−^ clones were sequenced using Sanger method. Chromatograms of the sequences were then analysed using Benchling (https://www.benchling.com/, accessed on 14 January 2021). Green peaks = Adenine, red peaks = Thymine, blue peaks = Cytosine, and black peaks = Guanine. Multiple peaks for one BP reveal that there are multiple sequences coming from different alleles, indicating mutation. BP mutations on the majority of sequences are shown with red boxes. (**A**) Chromatogram of the Sanger-sequenced BV-2 wild-type (WT) *Drd3*^+/+^ gene. (**B**) Chromatogram of the Sanger-sequenced *Drd3* gene on the CRISPR-Cas9 gene edited *Drd3*^−/−^ clone. (**C**) Murine *Drd3* gRNA sequence that was inserted into the Px458 plasmid is aligned with the chromatograms as a reference to identify the CRISPR-Cas9 cut site. (**D**) Chromatogram of the Sanger-sequenced BV-2 WT *Htr1a* gene. (**E**) Chromatogram of the Sanger-sequenced *Htr1a* gene on the *Htr1a*^−/−^ clone. (**F**) Murine *Htr1a* gRNA sequence that was inserted into the Px458 plasmid is aligned with the chromatograms as a reference point for the CRISPR-Cas9 cut site. (**G**) Indel spectrum produced by Tracking of Indels by Decomposition: TIDE analysis shows that in the Drd3^−/−^ clone, 38.5% of sequences had a deletion of 10 base pairs (BPs) and 42.1% of sequences had a deletion of 7 BPs. In the *Htr1a*^−/−^ clone, there were 38.6% of sequences with an insertion of 1 BP and 44.8% of sequences with a deletion of 2 BPs. (**H**,**I**) Western blots and (**J**) real-time qPCR data demonstrating negligible *Drd3* protein and gene expression *Drd3*^−/−^ cells. (**K**,**L**) Western blots and (**M**) real-time qPCR data demonstrating negligible *Htr1a* protein and gene expression *Htr1a*^−/−^ cells. Western blots were performed by resolving sample proteins using a gradient 4–20% Tris-glycine SDS-PAGE as previously reported. Bar graphs in I and L depict significantly reduced D3r and 5-HT1Ar protein expression in the *Drd3*^−/−^ and *Htr1a^−/−^* clones, respectively. Quantifications were performed using the ImageJ software, and normalised values were calculated by dividing the mean optical density of bands over the corresponding GAPDH. Data reported as mean ± SEM. Real-time qPCR amplifications were performed using selected primer optimised for qPCR analyses (<155 bp length), which recognise fragments within the coding sequence of the *Drd3* gene (for details refer to Table 2). Fold changes in each gene were obtained after normalisation to the endogenous reference gene (*S18*) and calculated using the comparative ΔΔCt method. *** *p* < 0.001, **** *p* < 0.0001 vs. wild-type as determined by using the unpaired *t* test. Drd3: murine dopamine D3 receptor gene, 5-HT1Ar: 5-hydroxytryptamine 1A receptor, Drd3.3: murine dopamine D3 receptor gene knockout clone number 3, Htr1a.2: murine 5-hydroxytryptamine 1A receptor gene knockout clone number 2, RC: reagent control, gRNA: guide RNA, Ref: reference, S18: ribosomal protein S18, GAPDH: Glyceraldehyde 3-phosphate dehydrogenase, kDa: Kilodalton, KO: knockout.

We generated stable Drd3^−/−^ and Htr1a^−/−^ cell lines in BV-2 microglial cells to study the pharmacological effects of buspirone following LPS challenge. BV-2 cells were transfected with one of two separate Px458 plasmids, one targeting the *Drd3* gene and the other targeting the *Htr1a* gene (please refer to Supplementary Figure S1 for details). Sanger sequencing analysis of genomic DNA (for sequencing primers details, please refer to Table 3) revealed insertions/deletions (indels) in transfected cells (Figure 1A–F). Sanger sequencing of the alleles of *Drd3^−/−^* BV2 cells showed the presence of deletions of either 7 base pairs (BPs) or 10 BPs (Figure 1G), and the alleles of *Htr1a^−/−^* cells demonstrated an insertion of 1 BP or a deletion of 2 BPs (Figure 1G). Amino acid translation from the sequencing data indicated that all CRISPR-Cas9-induced mutations resulted in early stop codons (Supplementary Figure S4). Western blot (Figure 1H,I) and real-time qPCR analyses (Figure 1J) in Drd3^−/−^ cells further confirmed the knockout of the Drd3 protein and gene (*** *p* < 0.0001). Similarly, the absence of the 5-HT1Ar protein and gene expression in *Htr1a^−/−^* cells was confirmed by Western blot (Figure 1K,L) and qPCR (Figure 1M, **** *p* < 0.0001). 

### 3.2. Establishing the Optimal Buspirone Concentration and Assessing the Effects of Drd3 and Htr1a Gene Deletions and Buspirone Treatment on Cell Viability Following Exposure to LPS 

To investigate the effects of targeted Drd3 and Htr1a gene deletions in BV2 microglial cell lines before and after LPS challenge, we first established the optimal concentration to rescue LPS-induced cell polarisation in the absence of overt toxicity. To do so, MTT and Griess reagent assays were conducted in wild-type BV-2 microglia stimulated with LPS (1 μg/mL) and increasing concentrations of buspirone (0, 100, 200, 300 and 400 µM) at 24 h. Thereafter, MTT assays were performed under the same experimental conditions in both wild-type, *Drd3*^−/−^ and *Htr1a*^−/−^ cells exposed to LPS or LPS + buspirone (300 μM), which was found to be the most effective concentration, devoid of toxic activity at the tested time-point.

**Figure 2:**
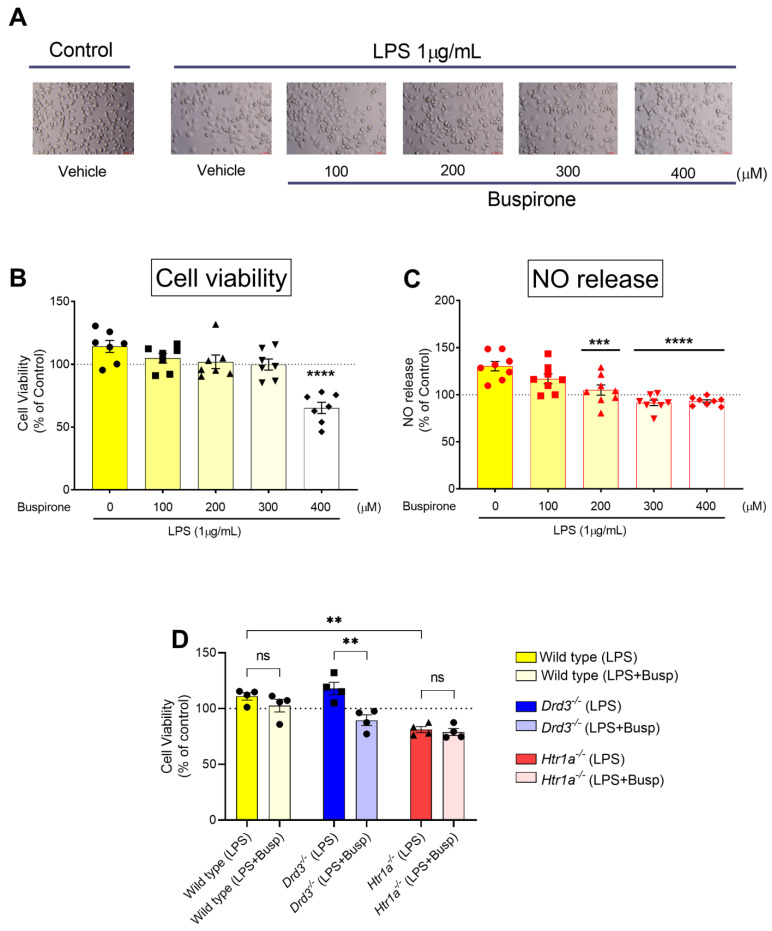
Effects of buspirone on cell viability in wild type, *Drd3*^−/−^ and *Htr1a*^−/−^ BV-2 microglial cells after co-treatment LPS and buspirone. Cells were either treated with LPS (1 μg/mL), alone or in combination with increasing concentrations of buspirone (100, 200, 300 and 400 µM, respectively) for 24 h and representative photomicrographs (using embossing filter settings) were taken (**A**). BV-2 cells treated as in A were tested for changes in cell viability (**B**) or nitric oxide release (**C**). Wild-type, *Drd3*^−/−^ and *Htr1a*^−/−^ BV-2 cells were treated with LPS and 300µM buspirone for 24 h before cell viability was assessed (**D**). Ns = not significant. Data reported is the mean ± SEM from at least two independent experiments. ** *p* < 0.01, *** *p* < 0.001, **** *p* < 0.0001 as determined by unpaired *t*-test (**A**–**C**) or two-way ANOVA followed by Tukey post-hoc test (**D**) Scale bar = 60 µm. WT: wild type, *Drd3*: murine dopamine D3 receptor gene, *Htr1a*: murine 5-hydroxytryptamine-1a receptor gene, LPS: lipopolysaccharide.

As shown in Figure 2A, LPS treatment induced obvious changes in microglial morphology (i.e., flattening and swelling of the soma). Co-treatment with increasing concentrations of buspirone had no significant effect on cell viability up to 400 µM, although cell viability dropped significantly (24.73% decrease, **** *p* < 0.0001 vs. LPS and/or untreated controls, Figure 2B). In terms of NO release (a pro-inflammatory mediator), LPS-induced NO release was partially rescued with 200µM buspirone (*** *p* < 0.001 vs. LPS); however complete recovery was seen at concentrations ≥300 µM buspirone (**** *p* < 0.0001 vs. LPS, Figure 2C). When comparing the effects of LPS and buspirone across the different genotypes, we observed some genotype-specific changes in cell viability in response to the inflammatory mimetic and the drug. Specifically, LPS significantly diminished cell viability in *Htr1a*^−/−^ cells (** *p* = 0.007), but not in wild-type or *Drd3*^−/−^ cultures (non-significant, *p* > 0.05) (Figure 2D). At the concentration tested, buspirone was not toxic to wild-type microglial cells (*p* = 0.4198) and did not further reduce viability in the *Htr1a*^−/−^ genotype (*p* = 0.6758), whereas it showed significant toxicity in LPS-treated *Drd3*^−/−^ cells (Figure 2D).

### 3.3. Nitric Oxide Release in Wild-Type, Drd3^−/−^ and Htr1a^−/−^ Microglial Cells Following LPS Challenge and Buspirone Treatment

**Figure 3:**
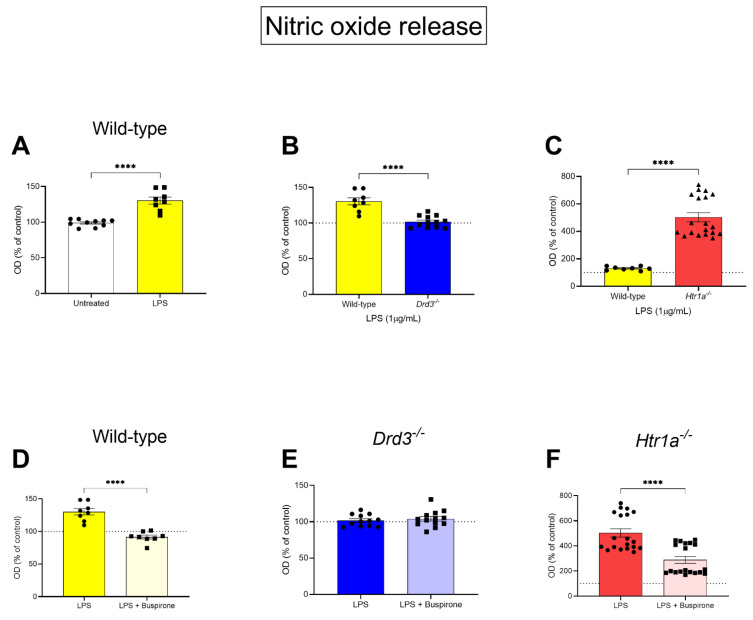
Nitrate levels in BV-2 wild type, *Drd3^−/−^* and *Htr1a^−/−^* cells after treatment with LPS in the presence of buspirone or not. Cells were treated for 24 h with either vehicle, 1 μg/mL LPS or 1 μg/mL LPS and 300 μM buspirone. Supernatants were then collected and placed in a new 96-well plate and incubated with Griess reagent for 15 min at room temperature, then read at 540 nm. Data shown are the optical density (OD), expressed as percentage of untreated WT cells (dotted lines). (**A**) Bar graph showing nitrate levels in wild-type BV-2 cells treated or not with LPS alone for 24 h. (**B**,**C**) Comparative assessment of NO release between (**B**) wild-type and *Drd3^−/−^* or (**C**) wild-type and *Htr1a^−/−^* cells after treatment with LPS. (**D**–**F**) Bar graphs depicting NO levels in the supernatants of (**D**) wild-type, (**E**) *Drd3^−/−^* and (**F**) *Htr1a^−/−^* cells after treatment with LPS and buspirone. Values are reported as the percentage NO release of untreated controls (dotted lines, except in **A**). Data reported as mean ± SEM. **** *p* < 0.0001 vs. LPS or untreated control, as determined by unpaired *t*-test.

To determine whether genetic deletion of the Drd3 or the Htr1a genes and/or buspirone treatment altered the responsiveness of BV2 microglia to an LPS challenge, we quantified the levels of nitric oxide (NO) released in supernatants, and measured relative levels using the Griess reagent assay. In WT cells, LPS significantly increased NO release (+30%, **** *p* < 0.0001, Figure 3A). When comparing the effects of LPS stimulation between wild-type vs. *Drd3^−/−^* cells, NO release was significantly reduced (**** *p* < 0.0001), reaching levels comparable to those seen in untreated wild-type controls (Figure 3B). Interestingly, LPS triggered an exaggerated NO release (about 5-fold that of WT cells) in the supernatant of *Htr1a^−/−^* cells (**** *p* < 0.0001) (Figure 3C). 

To determine the specific involvement of the Drd3 or the Htr1a in buspirone’s ability to mitigate LPS-driven NO release, we measured NO release after co-treatment with LPS and buspirone across the different genotypes. As shown in Figure 3D, buspirone reliably diminished the heightened NO levels in LPS-stimulated wild-type microglia (**** *p* < 0.0001). However, in Drd3^−/−^ cells, where LPS failed to induce additional NO release, buspirone was devoid of any biological activity (Figure 3E). Finally, Htr1a^−/−^ microglial cells also responded reliably to buspirone treatment (**** *p* < 0.0001), although NO levels were still around 2.5-fold that of wild-type controls (Figure 3F). 

### 3.4. Effects of Buspirone Treatment on Inducible Nitric Oxide Synthetase (NOS2) Gene Expression in Wild Type, Drd3^−/−^ and Htr1a^−/−^ Knockout Microglial Cells after Exposure to LPS 

In view of the efficacy of buspirone treatment in mitigating the NO induction triggered by LPS in BV-2 cells, we sought to determine if such beneficial effects were also associated with transcriptional changes in the mRNA expression of nitric oxide synthase 2 (*NOS2*), the gene encoding for inducible nitric oxide synthase (iNOS), an enzyme that catalyses the production of NO. 

**Figure 4:**
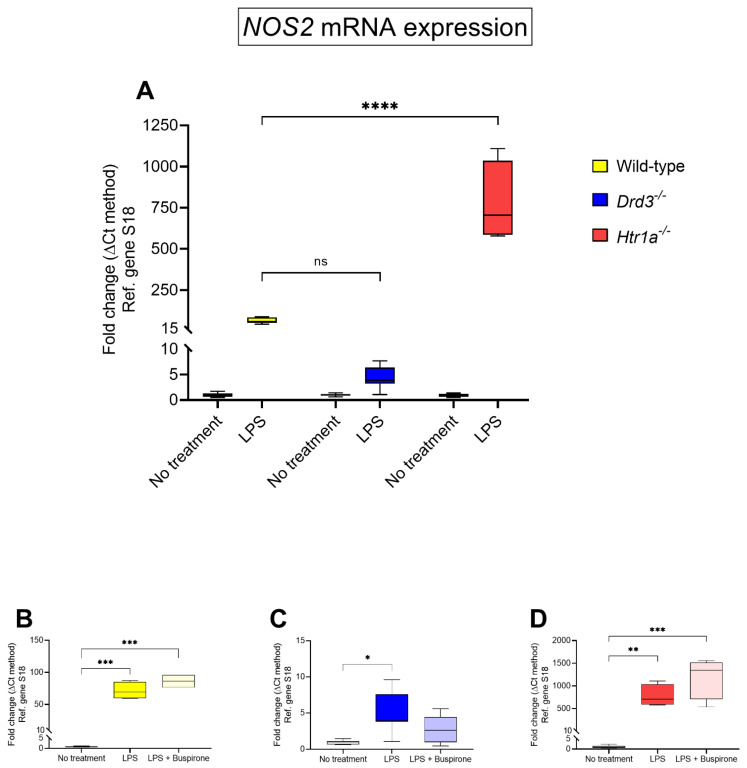
*NOS2* mRNA expression following exposure to LPS and treatment with buspirone for 24 h. Gene expression was measured using real-time qPCR and quantified using the ΔΔCt method after normalization to the S18 housekeeping gene. (**A**) Box and whisker plots showing the differential expression of *NOS2* transcripts in untreated or LPS-treated wild type, *Drd3^−/−^* and *Htr1a^−/−^* cells or after co-treatment of (**B**) wild type, (**C**) *Drd3^−/−^* and (**D**) *Htr1a^−/−^* cells with LPS in combination with buspirone. All the amplifications were performed using selected primers optimised for qPCR analyses (<155 bp length) which recognise fragments within the coding sequence of the gene of interest (for details please refer to Table 4). Results are presented as mean fold changes with respect to no treatment (control) ± SEM. Fold changes of each gene were obtained after normalisation to the endogenous reference gene. Baseline levels of no treatment groups were set to 1. Statistically significant data (* *p* < 0.05, ** *p* < 0.01, *** *p* < 0.001, **** *p* < 0.0001) were determined by one-way or two-way ANOVA followed by Tukey and Dunnett’s post-hoc tests. Ns = not significant.

Comparative analyses of *NOS2* transcripts across the different genotypes revealed some genotype-dependent changes in gene expression in response to LPS. Specifically, LPS-induced *NOS2* mRNAs were less pronounced in *Drd3^−/−^* microglia compared with wild-type (~4.3-fold in *Drd3^−/−^* vs. ~65-fold in wild-type cells, Figure 4A) although these changes were not statistically significant (*p* > 0.05, two-way ANOVA followed by Dunnett’s post-hoc test). In LPS-stimulated *Htr1a^−/−^* cells there was a remarkable increase in *NOS2* transcripts (**** *p* < 0.0001 vs. wild-type, Figure 4A).

When assessing the effects of buspirone within each genotype, buspirone failed to reduce LPS-driven increases in *NOS2* transcripts in wild-type cells (*p* > 0.05, Figure 4B). 

In *Drd3^−/−^* microglia, LPS effects on *NOS2* mRNA levels were blunted when compared to wild-type cells, but still significantly increased (* *p* < 0.05 vs. no treatment, Figure 4C). Treatment with buspirone reduced gene expression, resulting in levels that did not significantly differ from those of untreated cells (*p* = 0.3137 vs. no treatment in *Drd3^−/−^* cells, Figure 4C). By contrast, *NOS2* mRNA expression in LPS-exposed *Htr1a^−/−^* microglial showed robust increases (~782-folds vs. no treatment in *Htr1a^−/−^* microglia, ** *p* < 0.01) which further increased after buspirone treatment, although not at statistically significant levels (*p* = 0.07 vs. LPS, Figure 4D). 

### 3.5. Effects of Buspirone Treatment on Interleukin-1β (IL-1β) Gene Expression in Wild Type, Drd3^−/−^ and Htr1a^−/−^ Knockout Microglial Cells after Exposure to LPS

**Figure 5:**
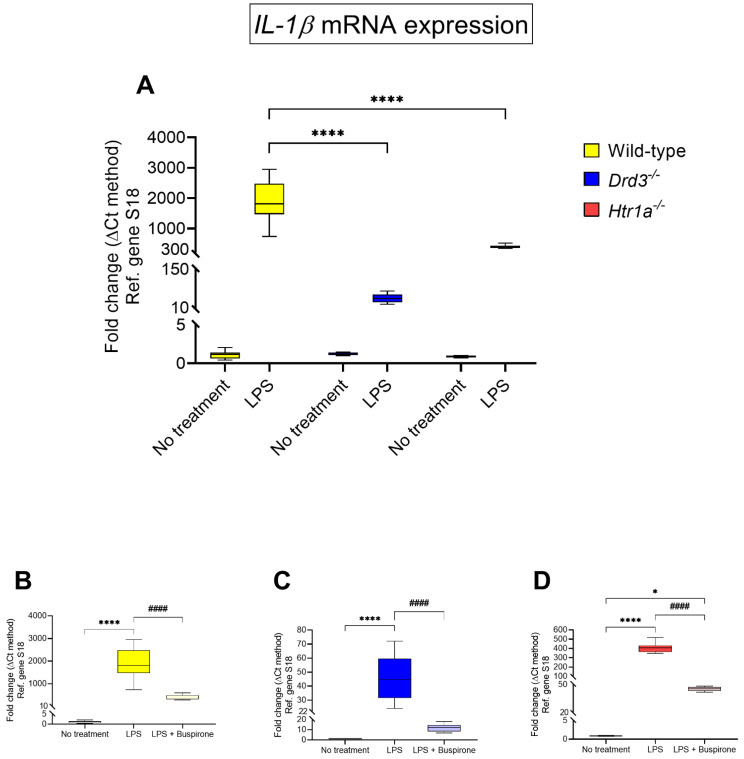
*IL-1β* mRNA expression following exposure to LPS and treatment with buspirone for 24 h. Gene expression was measured using real-time qPCR and quantified using the ΔΔCt method after normalization to the S18 housekeeping gene. (**A**) Box and whisker plots showing the differential expression of *IL-1β* transcripts in untreated or LPS-treated wild type, *Drd3^−/−^* and *Htr1a^−/−^* cells or after co-treatment of (**B**) wild type, (**C**) *Drd3^−/−^* and (**D**) *Htr1a^−/−^* cells with buspirone. Results are presented as mean fold changes with respect to no treatment (control) ± SEM. Baseline levels of no treatment groups were set to 1. * *p* < 0.05 or **** *p* < 0.0001 vs. no treatment or #### *p* < 0.0001 vs. LPS were determined by one-way or two-way ANOVA followed by Tukey and Dunnett’s post-hoc tests.

We used real-time qPCR to determine whether the mRNA expression of the pro-inflammatory marker interleukin-1β (*IL-1β*) differed across genotypes in BV-2 cells stimulated with LPS (Figure 5A) or within each genotype after combination treatment with LPS and buspirone (Figure 5B–D). 

*IL-1β* mRNA expression was notably higher than controls in wild-type cultures following LPS exposure (~1880-folds of untreated wild-type, **** *p* < 0.0001 vs. LPS wild-type, Figure 5A). 

Treatment with buspirone sharply reduced *IL-1β* mRNA expression (~380-fold of untreated wild-type, #### *p* < 0.0001 vs. LPS, Figure 5B), to levels that did not differ statistically to untreated wild-type controls (*p* = 0.2434, Figure 5B). 

In *Drd3^−/−^* cells, drug treatment strongly reduced LPS-driven *IL-1β* gene expression. (#### *p* < 0.0001 vs. LPS, Figure 5C).

Finally, the upregulated *IL-1β* mRNAs in LPS-stimulated *Htr1a^−/−^* microglial cells were partially reversed by buspirone treatment, although they were still elevated when compared with no treatment controls (* *p* < 0.05, Figure 5D).

### 3.6. Effects of Buspirone Treatment on Tumor Necrosis Factor-α (TNF-α) Gene Expression in Wild Type, Drd3^−/−^ and Htr1a^−/−^ Knockout Microglial Cells after Exposure to LPS

**Figure 6:**
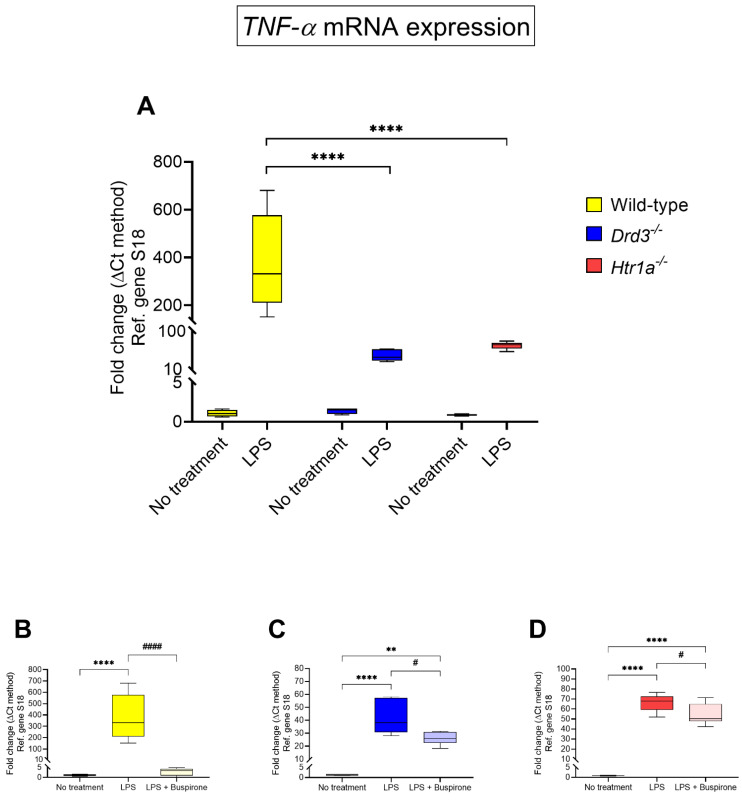
*TNF-α* mRNA expression following exposure to LPS and treatment with buspirone for 24 h. Gene expression was measured using real-time qPCR and quantified using the ΔΔCt method after normalization to the S18 housekeeping gene. (**A**) Box and whisker plots showing the differential expression of *TNF-α* transcripts in untreated or LPS-treated wild type, *Drd3^−/−^* and *Htr1a^−/−^* cells or after co-treatment of (**B**) wild type, (**C**) *Drd3^−/−^* and (**D**) *Htr1a^−/−^* cells with LPS in combination with buspirone. Results are presented as mean fold changes with respect to no treatment (control) ± SEM. Baseline levels of no treatment groups were set to 1. ** *p* < 0.01 or **** *p* < 0.0001 vs. no treatment or # *p* < 0.05 and #### *p* < 0.0001 vs. LPS, as determined by one-way or two-way ANOVA followed by Tukey or Dunnett’s post-hoc tests.

Among LPS treated BV-2 cells, we observed a remarkable increase in *TNF-α* mRNA expression (~377-fold increase vs. no treatment), the magnitude of which was not seen in either *Drd3^−/−^* or *Htr1a^−/−^* cells (~42-fold and ~65.82-fold, respectively), in which *TNF-α* gene expression was significantly reduced vs. wild-type (**** *p* < 0.0001 for both, Figure 6A). 

In wild-type BV-2 cells, buspirone potently diminished *TNF-α* transcripts (~2.87-folds of no treatment, #### *p* < 0.0001 vs. LPS, Figure 6B). By contrast, the drug only modestly reduced *TNF-α* gene expression in the *Drd3^−/−^* and *Htr1a^−/−^* cell lineages (# *p* < 0.05 vs. LPS for both genotypes, Figure 6C,D). 

### 3.7. Effects of Buspirone Treatment on Arginase 1 (Arg1) Gene Expression in Wild Type, Drd3^−/−^ and Htr1a^−/−^ Knockout Microglial Cells after Exposure to LPS

**Figure 7:**
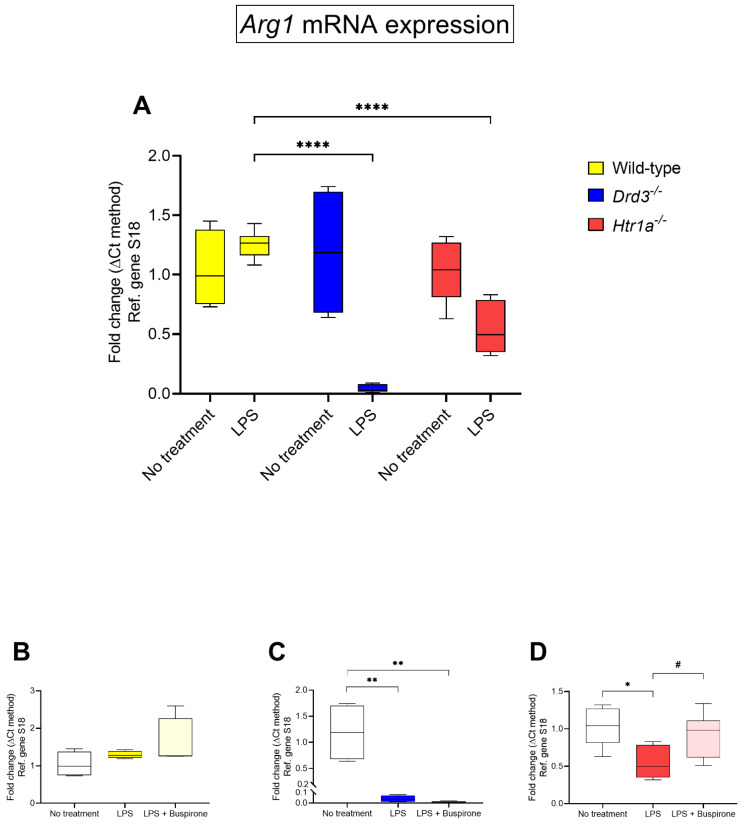
*Arg1* mRNA expression following exposure to LPS and treatment with buspirone for 24 h. Gene expression was measured using real-time qPCR and quantified using the ΔΔCt method after normalization to the S18 housekeeping gene. (**A**) Box and whisker plots showing the differential expression of *Arg1* transcripts in untreated or LPS-treated wild type, *Drd3^−/−^* and *Htr1a^−/−^* cells or after co-treatment of (**B**) wild type, (**C**) *Drd3^−/−^* and (**D**) *Htr1a^−/−^* cells with LPS in combination with buspirone. Results are presented as mean fold changes with respect to no treatment ± SEM. Baseline levels of no treatment groups were set to 1. * *p* < 0.05, ** *p* < 0.01 or **** *p* < 0.0001 vs. no treatment or # *p* < 0.05 vs. LPS, as determined by one-way or two-way ANOVA followed by Tukey or Dunnett’s post-hoc tests.

Comparative analyses of *Arg1* mRNA expression (an anti-inflammatory marker) across the different genotypes revealed significant differences of gene expression following LPS exposure (Figure 7A). Specifically, whereas in wild-type BV-2 cells, LPS failed to induce any significant changes in *Arg1* gene expression (*p* = 0.2963), there was a significant downregulation in *Drd3^−/−^* cells (**** *p* < 0.0001 vs. LPS-treated wild-type cells, Figure 7A) and, also to a lesser extent, in Htr1a^−/−^ cells (**** *p* < 0.0001, Figure 7B). 

In *Drd3^−/−^* cells, buspirone did not affect *Arg1* mRNA expression (*p* = 0.5993 vs. LPS-treated cells, Figure 7C), whereas in *Htr1a^−/−^* cells, buspirone treatment rescued the gene downregulation caused by LPS treatment (# *p* < 0.05 vs. LPS, Figure 7D). 

### 3.8. Effects of Buspirone Treatment on Found in Inflammatory Zone 1 (FIZZ1) Gene Expression in Wild Type, Drd3^−/−^ and Htr1a^−/−^ Knockout Microglial Cells after Exposure to LPS

To provide a balanced panel of both pro- and anti-inflammatory markers in our assessment of the activities of buspirone, we interrogated an additional microglial anti-inflammatory marker (i.e., found in inflammatory zone 1 gene, [*FIZZ1*]) across the different genotypes following exposure to LPS, and then within each genotype, to assess the effect of combined treatment with LPS and buspirone.

**Figure 8:**
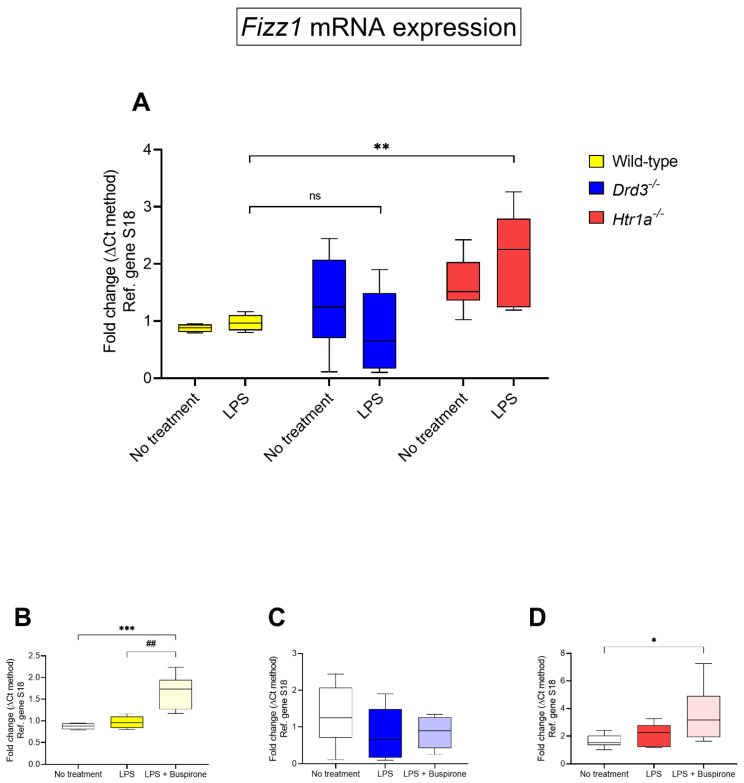
*FIZZ1* mRNA expression following exposure to LPS and treatment with buspirone for 24 h. Gene expression was measured using real-time qPCR and quantified using the ΔΔCt method after normalization to the S18 housekeeping gene. (**A**) Box and whisker plots showing the differential expression of *FIZZ1* transcripts in untreated or LPS-treated wild type, *Drd3^−/−^* and *Htr1a^−/−^* cells or after co-treatment of (**B**) wild type, (**C**) *Drd3^−/−^* and (**D**) *Htr1a^−/−^* cells with LPS in combination with buspirone. Results are presented as mean fold changes with respect to no treatment ± SEM. Fold changes in each gene were obtained after normalisation to the endogenous reference gene. Baseline levels of no treatment groups were set to 1. * *p* < 0.05, ** *p* < 0.01 or *** *p* < 0.001 vs. no treatment or ## *p* < 0.01 vs. LPS, as determined by one-way or two-way ANOVA followed by Tukey or Dunnett’s post-hoc tests. Ns = not significant.

When comparing LPS-stimulated wild-type vs. *Drd3^−/−^* cultures, there were no significant differences in *FIZZ1* gene expression (*p* = 0.7956 vs. LPS-treated wild-type cells, Figure 8A). However, we found a statistically significant increase in gene expression in *Htr1a^−/−^* cells (** *p* < 0.01, Figure 8A). Interestingly, buspirone significantly increased *FIZZ1* mRNA expression in LPS-stimulated, wild-type microglia (## *p* < 0.01 vs. LPS-treated wild-type cells, Figure 8B), but had no significant effect on LPS-treated *Drd3^−/−^* cultures (*p* = 0.9734, Figure 8C), whilst it triggered a moderate and significant gene upregulation in *Htr1a^−/−^* cells (* *p* < 0.05 vs. no treatment group, Figure 8D). 

## 4. Discussion

This is the first study, to the best of our knowledge, which describes the anti-inflammatory effects of buspirone in BV-2 microglial cells after LPS challenge and investigates the involvement of the genes for *Drd3* and *Htr1a* in mediating beneficial drug-induced effects. Buspirone has been available on the market for decades in view of its beneficial activities for the treatment of generalised anxiety disorder. Its central activity is still achieved despite low systemic bioavailability. In fact, when administered, buspirone is extensively metabolized due to its extensive first pass metabolism, where it undergoes hepatic oxidation mediated by the CYP3A4 enzyme. Hydroxylated derivatives are produced, including a pharmacologically active metabolite 1-pyrimidinylpiperazine (1-PP), which seems to participate in the biological activity of the drug [35]. Buspirone has a relatively low solubility and there is no documented evidence of active transport mechanisms through the blood brain barrier; nonetheless, as highlighted by an [11C]-(+)-PHNO occupancy study the drug reaches Drd3-rich CNS targets, where it exerts clear Drd3 antagonist activity [36]. These results pinpoint how buspirone may display some degree of permeability in the BBB. 

Using CRISPR-Cas9 technology, we selectively knocked out buspirone’s best-known pharmacological targets to create a robust in vitro platform to gain novel molecular insights into the mechanism of action of buspirone and other drugs targeting these receptor types. Our findings demonstrate that buspirone treatment and the genetic deletion of *Drd3* attenuates LPS-triggered inflammation in microglia cell lines. In addition, we have identified a novel biological role of the *Htr1a* gene in controlling microglial cell viability and *NOS2*-mediated nitric oxide production after LPS insult. 

### 4.1. Targeting Drd3 and Htr1a Genes to Study the Effects of Buspirone in Microglial Cells

A major reason for the generation of stable knockout BV-2 microglial cell lines using CRISPR-Cas9 gene editing technology was to develop a cellular platform that could help in unveiling the pharmacological activity of buspirone in microglial cells exposed to an inflammatory challenge with LPS. This approach was essential to overcome any issues associated with transient reduction of gene expression using either short-interfering RNA methods, or other gene knockdown strategies, which only attenuate gene expression for a limited time, and do not always recapitulate the biological effects achieved with total gene ablation with high fidelity. We have provided supplementary data detailing the general workflow (i.e., plasmid packaging, transfection, single-cell sorting of transfected cells and confirmation of gene deletions (Supplementary Figures S1–S4). Analyses of the mutations induced by CRISPR-Cas9 in clonal populations revealed the presence of insertion/deletions (indels) on up to four alleles instead of two. Interestingly, this phenomenon was reported in the same cell line after CRISPR-Cas9 editing of the gene *TREM2* [37]. Although the reasons why this occurs is not known, one possibility is the aneuploidy of the target chromosome(s) (i.e., tetrasomy). This would explain why CRISPR-Cas9 gene editing was particularly challenging in BV-2 cells in comparison to what would be expected from cells harboring the traditional bi-allelic configuration, as the chances of the CRISPR-Cas9 plasmid binding to all alleles are significantly reduced with twice the number of alleles. However, despite these challenges, we succeeded in generating complete gene knockout cell lines for each of the selected gene targets, providing a reliable and potentially scalable in vitro platform at a relatively low cost. Additionally, these protocols are now optimized and can be readily adapted to create a potent pharmacological tool to gain mechanistic insights on the pharmacology of both old and new drug targets [38]. 

Our CRISPR-Cas9 gene knockouts were confirmed both by Western blot and real-time qPCR. Frameshift mutations within the *Drd3* and *Htr1a* coding sequences were translated as truncated and non-functional proteins, whose expression was either strongly reduced or not detected (Figure 1H,I,K,L). Accordingly, *Drd3* and *Htr1a* mRNA studies confirmed negligible transcript levels in the two knockout cell lines (Figure 1J). It should be noted that some residual D3r protein expression was detected in the *Drd3^−/−^* microglia (Figure 1H,I). This can be explained by the fact that there are no commercially available antibodies that are specific to the D3r, as its amino acid sequence is highly homologous with that of other D2-like receptor subtypes, including the D2r and D4r [39], which also exhibit overlapping molecular weights. Therefore, the residual faint bands shown in *Drd3^−/−^* cells could be non-specific binding to any of these two receptors.

### 4.2. Genotype-Specific and Drug-Related Changes in BV-2 Cell Viability and Nitric Oxide Release after LPS Challenge 

Upon exposure to LPS, wild-type BV-2 microglia displayed signs of activation. However, buspirone dose-dependently reversed this process without significant toxic effects at concentrations up to 300 μM, which was also accompanied by a remarkable attenuation of nitrates in culture media (Figure 2). When comparing the effects of LPS and buspirone across the different genotypes, we found that exposure to LPS (1 μg/mL) slightly increased cell viability irrespective of genotype, surpassing levels of matched untreated controls, suggesting that the inflammatory mimetic might exert some growth-promoting effects. Similar results have previously been reported by another in vitro study that demonstrated an increase in the survival rate of BV-2 cells following exposure to the same concentration of LPS used in this study after exposures of 24 and 40 h [40]. In addition, our data are further supported by several in vivo studies that reported an increase in microglial cell proliferation in adult mice following a single dose of LPS [41,42]. 

With regards to BV-2 cell viability and nitric oxide release, our results using the *Htr1a^−/−^* cell line were totally unexpected. To the best of our knowledge, there are no reports on the effects mediated by the 5-HT1Ar on microglia. We found that baseline cell viability in *Htr1a^−/−^* microglia was significantly reduced compared with WT controls, both in LPS and cells treated with both LPS and buspirone. As there were no changes in viability resulting from the deletion of the *Drd3* gene, these findings suggest that the *Htr1a* might play a critical role in regulating the metabolic activity of these cells. A few studies have shown that microglia express functional serotonin receptors which are linked to distinct microglial properties and cellular interactions [43,44]. Our study provides unprecedented data supporting a functional role of the 5-HT1Ar in regulating BV-2 microglia cell viability. 

The degeneration of dopaminergic neurons has been linked to the robust upregulation of inducible nitric oxide synthase (NOS2) and production of neurotoxic levels of NO [45]. Our data show that *Htr1a* gene ablation in BV-2 cells exposed to LPS results in an exaggerated production of NO. These data were confirmed by analyses of *NOS2* mRNA expression, a gene that regulates the production of NO through the expression of *NOS2*. This result complements an earlier study by Zhang and collaborators [46], which found that the 5-HT1Ar agonist 8-OH-DPAT downregulated the neuronal-specific nitric oxide synthase (nNOS) expression and the 5-HT1Ar selective antagonist NAN-190 upregulated nNOS expression [46]. Based on these results, we are tempted to believe that stimulation of the 5-HT1Ar may hinder the activity of the NOS2/NO pathway, whilst gene ablation may do the opposite. Importantly, buspirone itself was able to decrease NO production in both WT and *Htr1a^−/−^* cells co-treated with LPS. 

This novel finding suggests that the decrease in NO levels seen after buspirone treatment is most likely due to the antagonist activity of the Drd3 and not the agonist function on 5-HT1Ar. This is further supported by the data obtained in *Drd3^−/−^* cultures, where stimulation with LPS failed to increase NO and only marginally increased *NOS2* gene expression, in alignment with the idea that Drd3 antagonism attenuates neuroinflammation [47,48]. 

### 4.3. Genotype-Specific and Drug-Related Changes of Pro-Inflammatory Cytokines in LPS-Stimulated BV-2 Microglia 

Both IL-1β and TNF-α have been implicated as main effectors of the neuroinflammatory machinery activated during neurodegeneration in models of PD [48]. Moreover, the exogenous administration of both cytokines has been shown to exacerbate 6-OHDA-induced cell death [48]. In this study, we first established whether LPS stimulation triggered the induction of IL-1β and TNF-α gene expression and then identified if there were specific differences in the expression profile of these pro-inflammatory across genotypes and in response to buspirone treatment. As expected, LPS challenge robustly increased the expression of both cytokines (in the order of about 2000-fold). In *Drd3^−/−^* and, to a lesser extent, in *Htr1a^−/−^* cells, LPS-driven IL-1β and TNF-α gene induction were much more blunted than in WT cells, providing additional evidence for a role of the *Drd3* (and perhaps the *Htr1a*) in mitigating LPS-induced inflammation. The latter consideration is in view of the effects seen after drug treatment in both genotypes, where buspirone further prevented the upregulation of both pro-inflammatory cytokines in both genotypes (*Drd3^−/−^* and *Htr1a^−/−^* BV-2 cells, respectively). This corroborates the findings in murine models, showing that either IL-1β or TNF-α induction are attenuated by xaliproden or 8-OH-DPAT, two 5-HT1Ar agonists [49,50]. However, the study highlights the predominant role of the Drd3 blockage as the main route to provide an anti-inflammatory activity in BV-2 microglia, which is supported further by evidence showing that Drd3 agonists trigger TNF-α gene upregulation [51]. 

### 4.4. Genotype-Specific and Drug-Related Changes of Anti-Inflammatory Cytokines in LPS-Stimulated BV-2 Microglia

In addition to pro-inflammatory cytokines, we also analysed the expression levels of the anti-inflammatory cytokines *Arg1* and *FIZZ1*. Our study showed that, in untreated cells, neither of these cytokines were affected across the genotypes (WT, *Drd3^−/−^*, *Htr1a*^−/−^). When cells were challenged with LPS, consistent with previous evidence using similar experimental paradigms [52,53,54], *FIZZ1* gene expression was unaffected, whereas Arg1 mRNAs were diminished in both *Drd3^−/−^* and *Htr1a*^−/−^ cells. Treatment with buspirone was unable to rescue *Arg1* gene downregulation in WT and *Drd3^−/−^* cells but was effective in restoring gene expression in *Htr1a*^−/−^ cells, suggesting a more complex interaction between the Drd3 and 5HT1ar in regulating *Arg1* transcription. In contrast, FIZZ1 mRNA expression was strongly increased in response to buspirone treatment in WT BV-2 cultures, but not in *Drd3^−/−^* cells and only marginally in *Htr1a^−/−^* cells. Taken together, this evidence supports the notion that, during an LPS challenge, the Drd3 antagonist activity of buspirone is required to convey its anti-inflammatory response. Similar to recent reports from Pacheco and co-workers in a PD model [14] and in a model of methamphetamine-induced intoxication [55], our findings reveal a critical role for Drd3 in modulating the inflammatory response. The upregulation of anti-inflammatory cytokines in microglia is considered critical in slowing the progression of PD and other neurodegenerative conditions, as this promotes the acquisition of the M2 anti-inflammatory phenotype by microglia [56]. PG01037, a potent Drd3 antagonist, reduced microglia activity, thereby contributing to an overall anti-inflammatory and therapeutic effect in PD mouse models [25]. 

Despite the promising results of this study, it is important to note that microglia are not the only cells contributing to the chronic inflammatory milieu in the CNS of people afflicted by neurological disorders. Astrocytes constitute the majority of the brain’s glial cell population and communicate closely with microglia to respond to neuronal damage, inflammatory and pathological stimuli. Montoya and colleagues [14] demonstrated that the Drd3s are expressed on astrocytes and that Drd3 deficiency in astrocytes attenuates microglial activation upon systemic inflammation and exacerbated the expression of the anti-inflammatory *FIZZ1* gene [14]. Furthermore, the lack of Drd3 expression in astrocytes promotes beneficial astrogliosis, which exerts anti-inflammatory and neuroprotective functions [25]. These beneficial actions of Drd3 blockade on astrocytes are particularly important in neurological diseases associated with neuroinflammation as it has been shown that, during chronic neuroinflammation, astrocytes alter the permeability of the blood brain barrier to promote the infiltration of peripheral immune cells to further promote neuroinflammation. These studies suggest that buspirone’s anti-inflammatory activities may not be restricted to microglia, and that its Drd3 antagonist activity on astrocyte might elicit synergistic effects via alternative mechanisms that culminate in a robust anti-inflammatory effect that also promotes neuroprotection. 

## 5. Conclusions

In conclusion, in the present manuscript, we show that either *Drd3* gene deletion or buspirone treatment reliably attenuated LPS-triggered inflammation in BV-2 microglia. Additionally, we are the first to demonstrate the functional role of the 5-HT1Ar in regulating cell viability and NO release in polarised microglia. The ability of both the *Drd3* gene deletion and buspirone treatment and, to a lesser extent, *Htr1a* gene ablation, to reduce inflammation in microglia suggests that buspirone holds the potential to be repurposed as an effective anti-inflammatory agent to treat those neurological diseases associated with chronic neuroinflammation, including Parkinson’s disease. In addition, considering that there is some evidence suggesting that buspirone may also exert stimulatory activities in immune cells of the periphery, such as CD4+ T lymphocytes [57] and natural killer cells [58], in vivo work is warranted to better elucidate the full potential of this drug in the CNS.

## Figures and Tables

**Table 1:** Guide RNA (gRNA) primers used to target the mouse *Drd3* and *Htr1a* genes with the CRISPR-Cas9 plasmid.

Gene (Ref. Seq.)	Forward Sequence 5′-3′ Reverse Sequence 3′-5′	Location	Tm (°C)
Mouse *Drd3* (NC_0.000082.6)	(AAAC) CATGCCTACTACGCCCTGTC (CACC) GACAGGGCGTAGTAGGCATG	472 491	69.7 73.9
Mouse *Htr1a* (NC_0.000079.6)	(CACC) GGTGCTCGGCAATGCCTGCG (AAAC) CGCAGGCATTGCCGAGCACC	501 520	84.2 80.0

**Table 2:** PCR primers used to amplify the mouse *Drd3* and *Htr1a* genes.

Gene (Ref. Seq.)	Forward Sequence 5′-3′ Reverse Sequence 3′-5′	Location	Tm (°C)	Length (BP)
Mouse *Drd3* (NC_0.000082.6)	TTGTTGTCTGTGTTCGCCCA AGGGTCCCATCATTCATGCC	23 883	67.8 68.2	861
Mouse *Htr1a* (NC_0.000079.6)	AGTGAAATGGACAGCGCGA AATGAGCCAAGTGAGCGAGA	27 840	67 65.1	814

**Table 3:** Primers used for the sequencing of the mouse *Drd3* and *Htr1a* genes.

Gene (Ref. Seq.)	Forward Sequence 5′-3′ Reverse Sequence 3′-5′	Location	Tm (°C)	Length (BP)
Mouse *Drd3* (NC_0.000082.6)	CCAGTTCTTACAGCACTGCCT CCGGAGCAGCATGTACCATAA	209 688	63.3 66.9	480
Mouse *Htr1a* (NC_0.000079.6)	CCCTTCGAAACTCCCCAGAAA GAGCCGATGAGATAGTTGGCA	239 581	68.2 66.4	343

**Table 4:** List of primers sets used in real-time quantitative PCR analyses.

Gene	GeneBank Accession Number	Primer Sequence	Length (bp)
S18	NM_011296.2	Fwd 5′ CCCTGAGAAGTTCCAGCACA 3′ Rev 5′ GGTGAGGTCGATGTCTGCTT 3′	145
Drd3	NM_007877	Fwd 5′ GGGGTGACTGTCCTGGTCTA 3′ Rev 5′ AAGCCAGGTCTGATGCTGAT 3′	100
IL-1β	NM_008361.4	Fwd 5′ GCTACCTGTGTCTTTCCCGT 3′ Rev 5′ CATCTCGGAGCCTGTAGTGC 3′	164
TNF-α	NM_013693.3	Fwd 5′ ATGGCCTCCCTCTCATCAGT 3′ Rev 5′ TTTGCTACGACGTGGGCTAC 3′	97
FIZZ1	NM_020509.3	Fwd 5′ AGCTGATGGTCCCAGTGAAT 3′ Rev 5′ AGTGGAGGGATAGTTAGCTGG 3′	98
Arg1	NM_007482.3	Fwd 5′ ACAAGACAGGGCTCCTTTCAG 3′ Rev 5′ TTAAAGCCACTGCCGTGTTC 3′	105
NOS2	NM_010927.4	Fwd 5′ TACCAAAGTGACCTGAAAGAGG 3′ Rev 5′ TCATCTTGTATTGTTGGGCTGA 3′	89

Forward and reverse primers were selected from the 5′ and 3′ region of each gene mRNA. The expected length of each amplicon is indicated in the right column.

## Data Availability

The data presented in this study are available on request from the corresponding author.

## Supplementary Materials

The following are available online at https://www.mdpi.com/article/10.3390/cells10061312/s1, Figure S1: Design of the Px458 plasmid for the murine Drd3 and Htr1a gene KOs and inserted their gRNA sequences. (**A**) General design of the Px458 plasmid, depicting the key features: human U6 promoter, gRNA sequences which were designed to bind specifically to the DNA of target genes, Cas9 protein, GFP, and ampicillin resistance. (**B**,**C**) Chromatograms created in Benchling from Sanger sequencing results and aligned gRNA sequences. Green peaks indicate an Adenine base, red peaks indicate a Thymine base, blue peaks indicate a Cytosine base and black peaks indicate a Guanine base. (**B**) Sequencing of the Drd3 plasmid indicates an incorrect BP of Guanine instead of Cytosine represented by the red box, however, there is still a blue peak for Cytosine, so this is likely sequencing error. (**C**) Sequencing results of the Htr1a plasmid shows correct alignment of the gRNA sequence. GFP: green fluorescent protein, gRNA: guide RNA, BP: base pairs, CRISPR: clustered, regularly interspaced short palindromic repeats. Drd3: murine dopamine D3 receptor gene, Htr1a: murine 5-hydroxytryptamine-1a receptor gene. Figure S2: Images of transfected BV-2 cells and fluorescence scatter dot plots displaying the gating of the GFP positive cells. Cells were seeded 1 × 10^5^ cells per well in 12-well plates and transfected with the appropriate plasmids. (**A**–**C**) Images of cells captured on a light microscope the day after transfection. (**D**–**F**) Images of cells under a light microscope with a GFP filter taken the day after transfection to illustrate the expression of GFP (shown by white arrow heads). (**G**–**I**) Each dot represents a single cell passing through the Fluorescence Activated Cell Sorting MoFlo© Astrios flow cytometer for sorting. The parameter for the horizontal axis of the dot plot is a Bandpass filter of 530/40 with a laser of 488 nm. The parameter for the vertical axis is a 750 longpass (LP) filter. (**G**) Dot plot of the BV-2 WT cells illustrates the GFP negative cells and therefore acts as a guide for gating the GFP positive cells as demonstrated with the orange box. (**H**,**I**) Dot plots of the BV-2 cells transfected with the CRISPR-Cas9 plasmids display the GFP positive cells gated inside the orange box. GFP: green fluorescent protein, WT: Wild type, KO: knockout, Drd3: murine dopamine D3 receptor gene, Htr1a: murine 5-hydroxytryptamine-1a receptor gene. Figure S3: DNA agarose gel electrophoresis (AGE) confirms that end-point PCR amplified Drd3 and Htr1a genes correctly. Cells were seeded 3 × 10^5^ cells per well in a 6-well plate, and DNA was extracted using ISOLATE II Genomic DNA Kit (Bioline). End point PCR was conducted using primers to target the genes of interest (for details refer to Table 2), and AGE confirmed the PCR was successful. Lane 1 is the DNA ladder (Hyperladder 1KB). Lane 2 is end-point PCR product of the Drd3 gene from BV-2 cells. Lane 3 is end-point PCR product of the Htr1a gene from BV-2 cells. BP: base pair, Drd3: murine dopamine D3 receptor gene, Htr1a: murine 5-hydroxytryptamine-1a receptor gene. Figure S4: DNA sequence and amino acid translation showing early stop codons in the Drd3^−/−^ and Htr1a^−/−^. End-point PCR products were Sanger sequenced and analysed through the program Tracking of Indels by Decomposition: TIDE. Amino acid translation was undertaken using Benchling. The corresponding amino acids for each codon are illustrated by coloured segments underneath the BPs in the DNA sequence. The number above each amino acid is indicative of the amino acid’s position in the aligned sequence in Benchling. The amino acids representing the stop codons are represented by the star symbol and are sur-rounded by red circles. Indels are illustrated by red boxes around the affected BPs. (**A**) Amino acid translation from Sanger se-quencing results of the Drd3^−/−^ indicates a deletion of 7 base pairs (BPs) and a deletion of 10 BPs. These resulted in frame-shift mu-tations and thus a change in the codons and the corresponding amino acid sequence compared to the WT. (**B**) Amino acid transla-tion from Sanger sequencing results of Htr1a^−/−^ reveal an insertion of 1 BP and deletion of 2 BPs, also resulting in frameshift muta-tions. WT: Wild type, A: adenine, T: thymine, C: cytosine, G: guanine. Drd3: murine dopamine D3 receptor gene, Htr1a: murine 5-hydroxytryptamine-1a receptor gene.
