# Supplementary figures and images for: Assessing the Anti-Inflammatory Activity of the Anxiolytic Drug Buspirone Using CRISPR-Cas9 Gene Editing in LPS-Stimulated BV-2 Microglial Cells
Source: Cells. 2021 May 25;10(6):1312. doi: 10.3390/cells10061312 (PMC8229595; doi:10.3390/cells10061312)

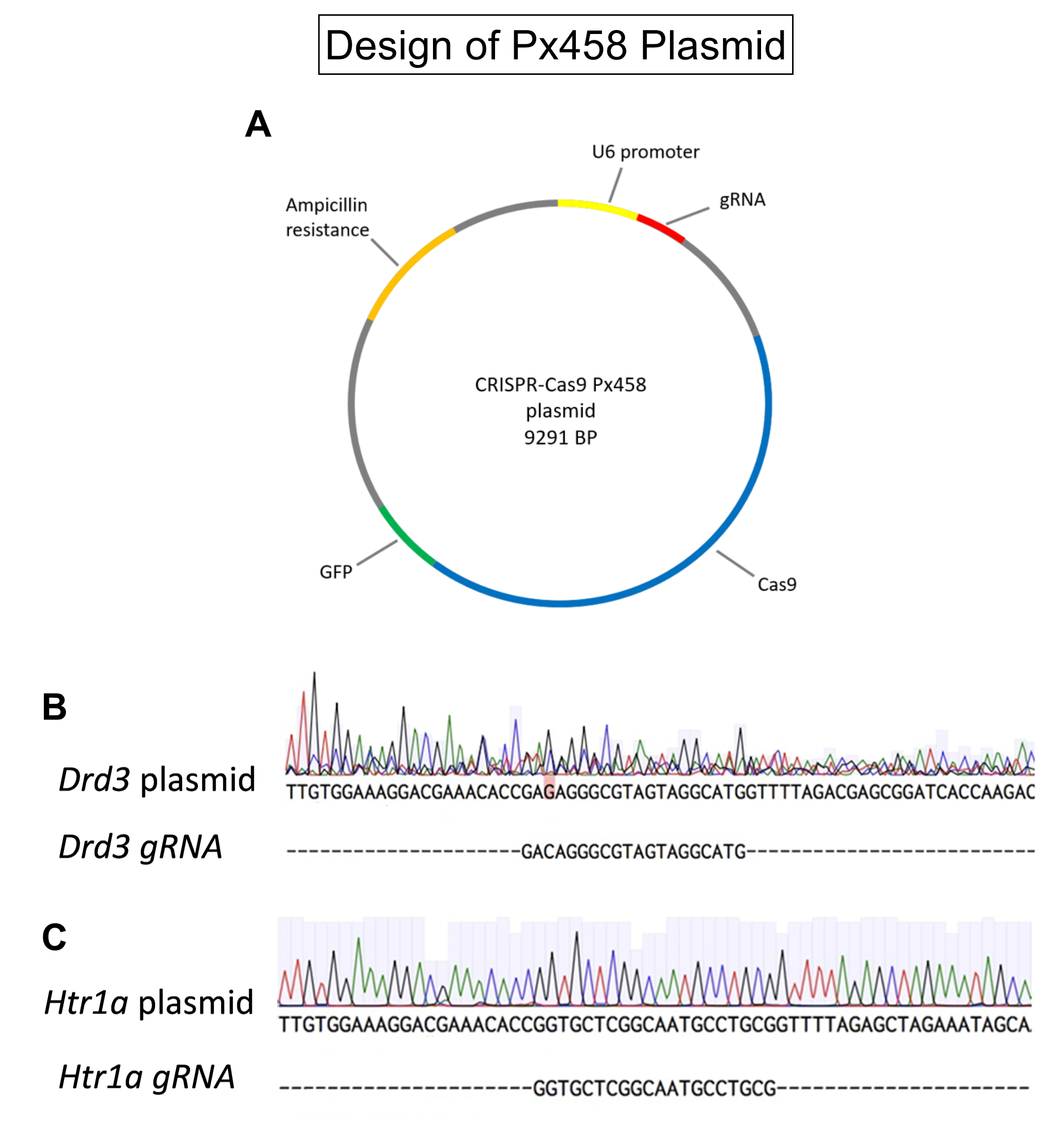

Supplement: Supplementary file 1 [file cells-10-01312-s001.zip › Supplementary Figure S1A-C (plasmid and gRNA design).tif]

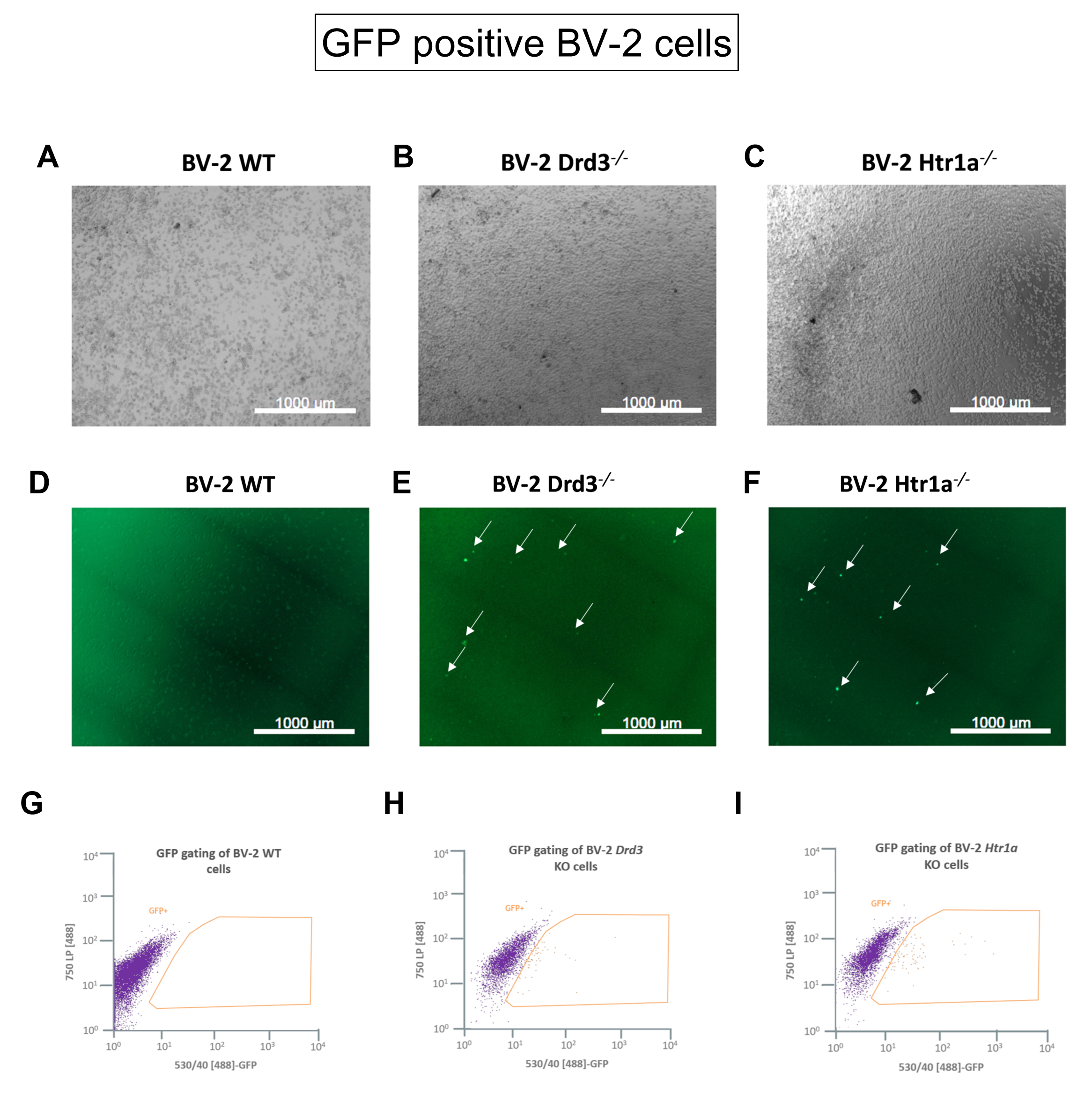

Supplement: Supplementary file 1 [file cells-10-01312-s001.zip › Supplementary Figure S2A-C (Single-cell sorting of GFP positive KO cell lines).tif]

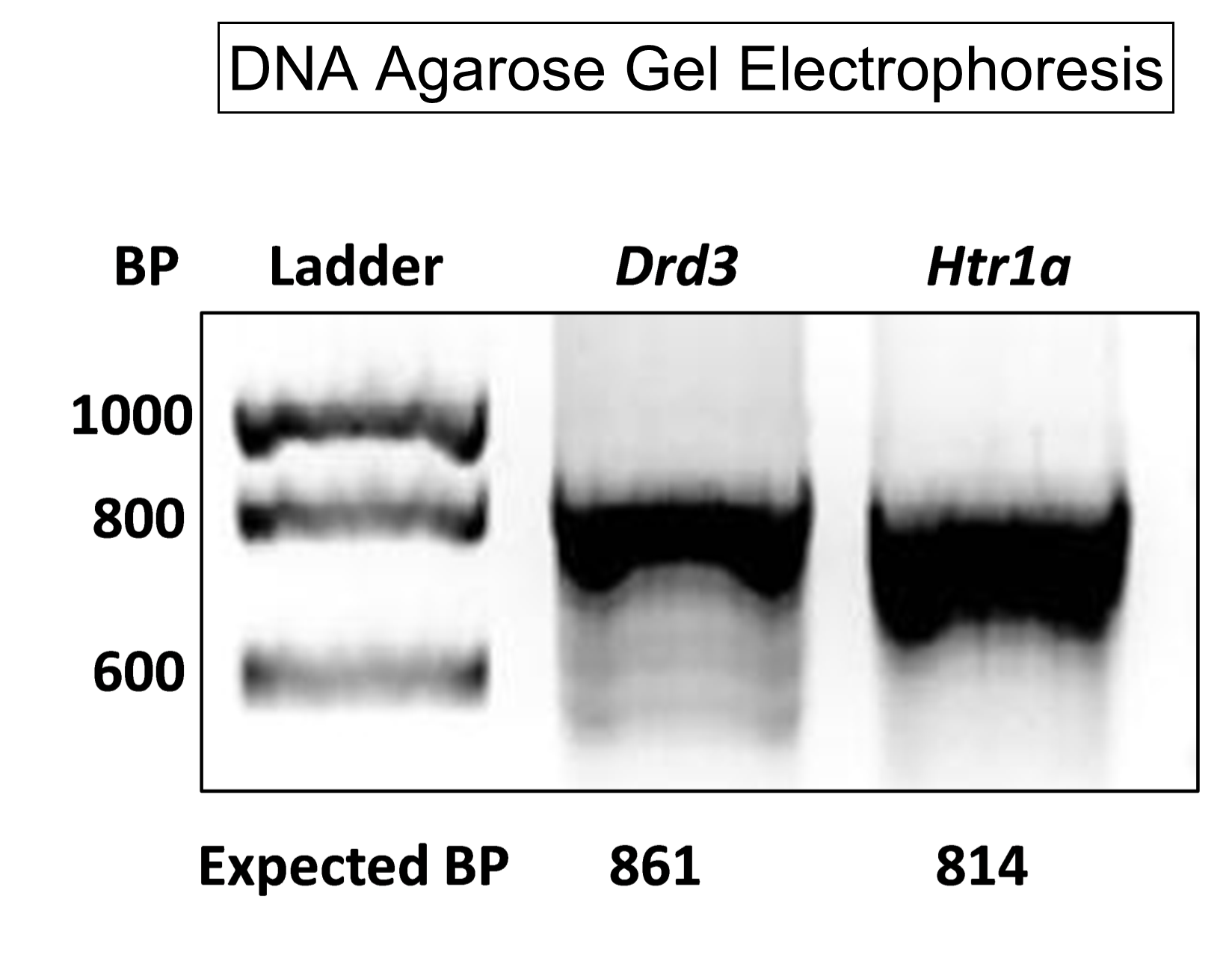

Supplement: Supplementary file 1 [file cells-10-01312-s001.zip › Supplementary Figure S3.tif]

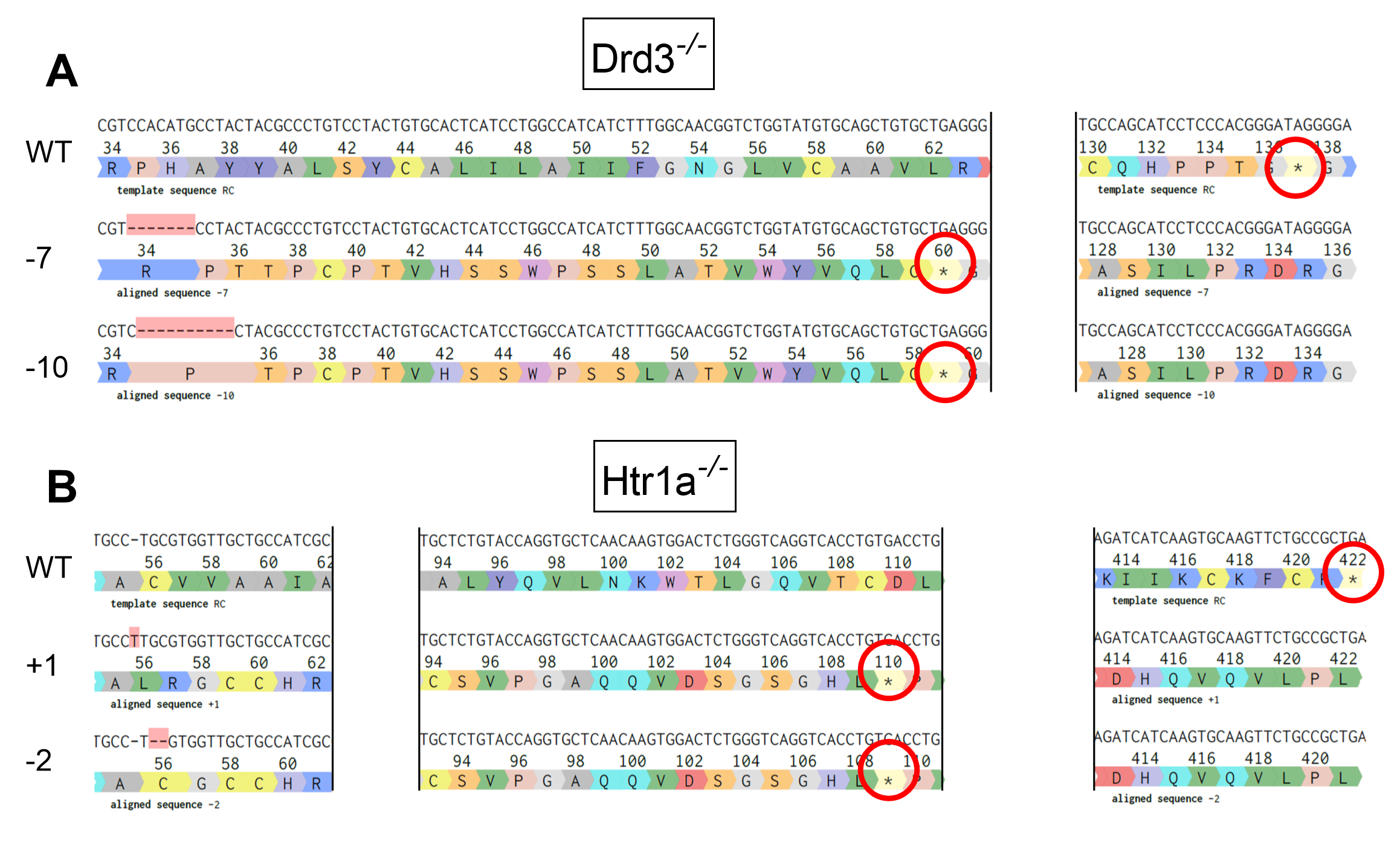

Supplement: Supplementary file 1 [file cells-10-01312-s001.zip › Supplementary Figure S4.tif]
